# Zebrafish Embryo Infection Model to Investigate *Pseudomonas aeruginosa* Interaction With Innate Immunity and Validate New Therapeutics

**DOI:** 10.3389/fcimb.2021.745851

**Published:** 2021-09-30

**Authors:** Stéphane Pont, Anne-Béatrice Blanc-Potard

**Affiliations:** ^1^ Laboratory of Pathogen-Host Interactions (LPHI), Université Montpellier, Montpellier, France; ^2^ CNRS, UMR5235, Montpellier, France

**Keywords:** *Pseudomonas aeruginosa*, zebrafish, innate immunity, host–pathogen interactions, drug screening

## Abstract

The opportunistic human pathogen *Pseudomonas aeruginosa* is responsible for a variety of acute infections and is a major cause of mortality in chronically infected patients with cystic fibrosis (CF). Considering the intrinsic and acquired resistance of *P. aeruginosa* to currently used antibiotics, new therapeutic strategies against this pathogen are urgently needed. Whereas virulence factors of *P. aeruginosa* are well characterized, the interplay between *P. aeruginosa* and the innate immune response during infection remains unclear. Zebrafish embryo is now firmly established as a potent vertebrate model for the study of infectious human diseases, due to strong similarities of its innate immune system with that of humans and the unprecedented possibilities of non-invasive real-time imaging. This model has been successfully developed to investigate the contribution of bacterial and host factors involved in *P. aeruginosa* pathogenesis, as well as rapidly assess the efficacy of anti-*Pseudomonas* molecules. Importantly, zebrafish embryo appears as the state-of-the-art model to address *in vivo* the contribution of innate immunity in the outcome of *P. aeruginosa* infection. Of interest, is the finding that the zebrafish encodes a CFTR channel closely related to human CFTR, which allowed to develop a model to address *P. aeruginosa* pathogenesis, innate immune response, and treatment evaluation in a CF context.

## Introduction

*Pseudomonas aeruginosa* is a mesophilic Gram-negative bacterium able to thrive in very diverse habitats, which is linked to its metabolic versatility, impressive number of regulators, and two-component systems (Arai, 2011). *P. aeruginosa* can colonize a broad range of hosts, from plants to animals including humans (de Bentzmann and Plesiat, 2011), where it is considered as an opportunistic pathogen as it mainly affects compromised patients. This pathogen is a leading cause of nosocomial contaminations capable of causing a myriad of infection types like bacteremia, pneumonia, keratitis, wound, and urinary tract infection, in immunodeficient subjects (e.g., neutropenic, burn, or oncological patients). In addition, most individuals with cystic fibrosis (CF), a genetic disorder caused by mutations of the cystic fibrosis transmembrane conductance regulator (CFTR) gene, are colonized by *P. aeruginosa*, which is a major cause of morbidity and mortality in these patients. In this autosomal recessive disease, the function of the chloride channel CFTR is impaired, leading to thick mucus in the lung, providing an environment particularly favorable for *P. aeruginosa* multiplication. CFTR also contributes to the innate immune response, being involved in the bactericidal activity of macrophages (Di et al., 2006; Zhang et al., 2010; Del Porto et al., 2011).

During acute infection, *P. aeruginosa* relies on a wide array of virulence factors, allowing the bacterium to move (flagella), adhere on host cells (pili), degrade host factors (secreted proteases), evade innate immune response, or transmigrate within the organism (Gellatly and Hancock, 2013; Klockgether and Tummler, 2017). Among these weapons, the type three secretion system (T3SS) contributes tremendously to virulence (Hauser, 2009). This nano-syringe allows *P. aeruginosa* to intoxicate various cell types through the injection of bacterial effectors into host cells. Despite being considered an extracellular pathogen, recent studies have emphasized that *P. aeruginosa* can enter host cells *in vivo* (Belon et al., 2015; Mittal et al., 2016; Kroken et al., 2018), resulting in a transient phase of intracellular residence, where T3SS also plays a role (Garai et al., 2019). Intracellular residence during infection is an important strategy for bacterial pathogens to hide from the immune system and antibiotics (Lamberti and Surmann, 2021). During long-lasting infections in CF lungs, *P. aeruginosa* is able to shift from a colonizing toward a persistent state, associated with the formation of biofilm, which is extremely hard to eradicate since it protects *P. aeruginosa* against host immune defenses and antibiotics (Faure et al., 2018). Biofilm formation relies on active quorum sensing (QS), a system allowing bacteria to communicate with each other thanks to the release of small signaling molecules.

*Pseudomonas aeruginosa* is known to be highly resistant to many currently used antibiotics, due to efflux systems expelling intracellular antibiotics, its low outer membrane permeability, and antibiotic-inactivating enzymes (Pang et al., 2019). The recent rising of multidrug-resistant isolates and the major threat they represent at the hospital have led the World Health Organization to classify *P. aeruginosa* as a critical priority for which there is an urgent need for new treatments (Tacconelli et al., 2018). In this line, anti-virulence strategies and phage therapy represent appealing alternatives that are currently being developed to complement antibiotic treatments (Muhlen and Dersch, 2016; Broncano-Lavado et al., 2021).

To investigate *P. aeruginosa* pathogenesis, a plethora of *in vivo* infection models have been used, ranging from mammals to non-mammalian animals like *Caenorhabditis elegans* or even plants (Rahme et al., 1997; Lorenz et al., 2016; Kaito et al., 2020). Zebrafish (*Danio rerio*) is a non-mammalian vertebrate model that is gaining increasing interest in infection studies as it fulfills the advantages of both mammalian and invertebrate models. Zebrafish have a number of advantages, in terms of methodological, financial, and ethical issues over mammalians models, and have been widely used as a model for studying host–pathogen interactions (Torraca and Mostowy, 2018). As a vertebrate, zebrafish is genetically and physically closer to humans than invertebrate models and has an innate immune system closely related to that of humans. In this review, we will focus on the embryo, which is increasingly considered for modeling human infections, including lung diseases, caused by bacterial pathogens (Hernandez et al., 2015; Gomes and Mostowy, 2020). Importantly, the optical transparency of the embryonic stages provides unprecedented opportunities to visualize bacterial infections in real time. Moreover, genetic tools are available to transiently knock down host genes through the generation of morphants using translation-blocking antisense oligonucleotides injected at the one-cell stage or permanently knock down host genes through the generation of mutated fish lines.

During the embryonic stage, only the innate immune system is functional (Masud et al., 2017), making embryos a model of choice to investigate the contribution of innate immune cells upon infection (Torraca et al., 2014). The zebrafish infection model has been used to investigate the involvement of macrophages and neutrophils, which are highly migratory cells, capable of phagocytosis and the subsequent killing of pathogens (Linnerz and Hall, 2020; Rosowski, 2020). Macrophages are the first immune cells to develop in a zebrafish embryo, as early as 22 h post-fertilization (hpf), and both functional macrophages and neutrophils are present by 30 hpf. Transgenic zebrafish lines with fluorescent macrophages or neutrophils have been generated to visualize specifically the interaction of pathogenic bacteria with these cell lineages. Reporter fish lines can also be generated to monitor the activation of the proinflammatory response *in vivo*. Moreover, chemical and genetic tools are available to selectively deplete macrophages or neutrophils and investigate their specific role during infection in zebrafish embryos (Rosowski, 2020).

The zebrafish embryo model has been used for the first time to follow *P. aeruginosa* infections and assess its virulence in 2009 by three different groups (Brannon et al., 2009; Clatworthy et al., 2009; Llamas et al., 2009), and detailed methodology for this novel model of infection has been reported (Llamas and van der Sar, 2014). We review here for the first time the various studies implying *P. aeruginosa* carried out in this novel infection model. More specifically, this subject has relevance in addressing *P. aeruginosa* pathogenesis, monitoring interaction with innate immune cells, and evaluating the efficacy of anti-infectious treatments. In addition, the interest of zebrafish embryo as CF model is discussed and future promising studies using this model are underlined.

## Infection Routes for *Pseudomonas aeruginosa* in Zebrafish Embryos

**Figure 1:**
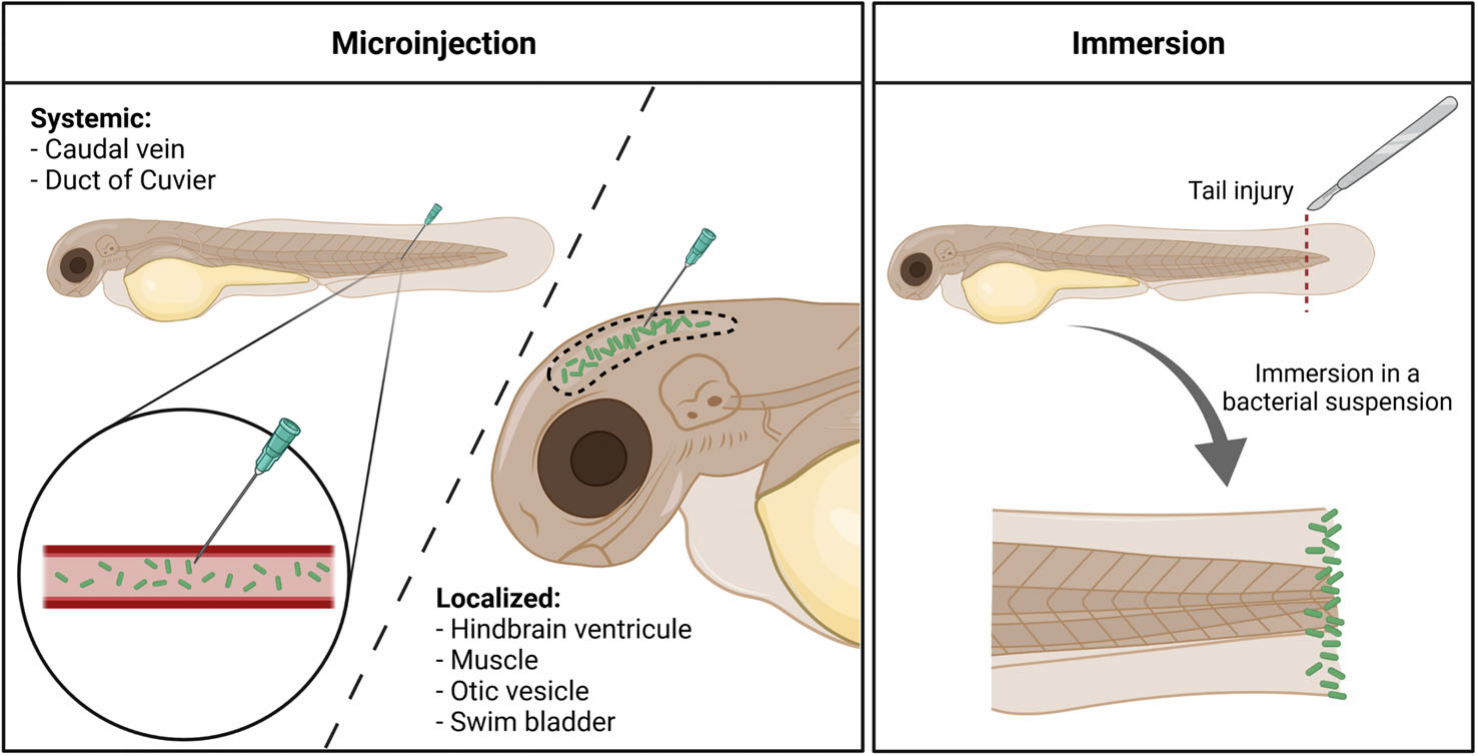
Different infection routes used with *Pseudomonas aeruginosa* in the zebrafish embryo. Microinjection offers the possibility to induce either a systemic invasion, by delivering bacteria (in green) directly within the circulation, or a more localized infection, by injecting the pathogen in closed compartments. Immersion of tail-injured embryos in a bacterial bath induces the colonization of the fish tail fin, mimicking a wound infection. Created with BioRender.com.

Infection in zebrafish embryos is most often established through microinjection of bacteria into the embryo, either in the circulation or in closed compartments (**Figure 1**). The site of microinjection determines whether the infection will rapidly become systemic or will initially remain localized. In addition, a bath immersion method has been developed with *P. aeruginosa* (**Figure 1**).

### Microinjection in the Circulation or in Closed Compartments

In initial experiments, *P. aeruginosa* infections were established by microinjecting bacteria into the bloodstream (injection in caudal vein or duct of Cuvier) of 1 or 2 days post-fertilization (dpf) old embryos, which causes a rapid systemic infection. The detailed handling for injection in the caudal vein has been described (Llamas and van der Sar, 2014).

**Figure 2:**
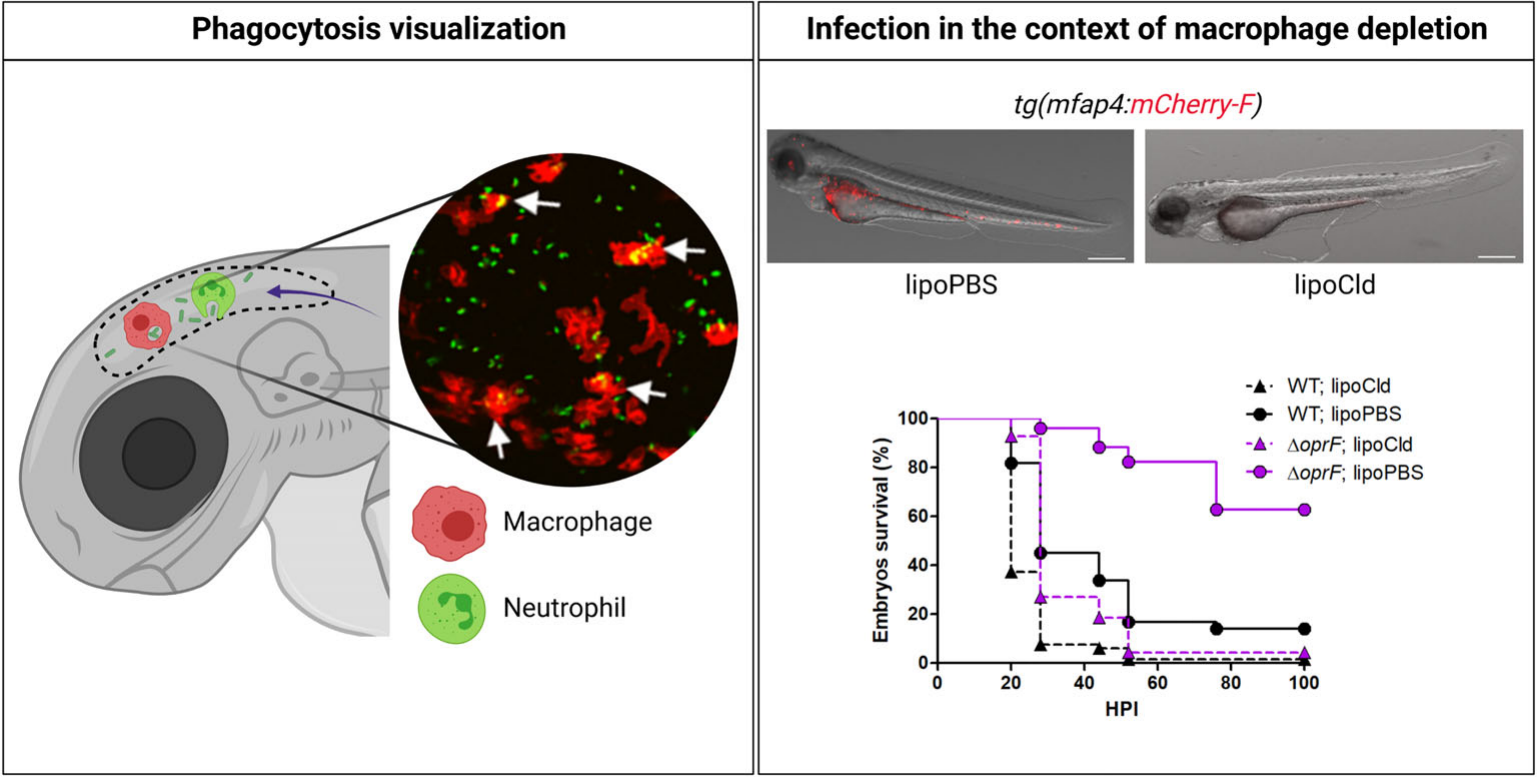
Advantages of zebrafish embryo to assess *in vivo* the interplay of *Pseudomonas aeruginosa* with innate immune cells. As shown in the left panel, using specific transgenic lines carrying fluorescently labeled macrophages [e.g., *tg(mfap4:mCherry-F)*] or neutrophils [e.g., *tg(mpx:GFP)*], and thanks to the transparency of the embryo, migration of professional phagocytes toward infectious foci (here HBV) and phagocytosed bacteria can be visualized in real time. The inserted picture is a confocal microscopy image where macrophages express mCherry (red labeling), *P. aeruginosa* PAO1 expresses GFP (green labeling), and phagocytosed bacteria are indicated by arrows (Moussouni et al., 2021). To assess the respective involvement of macrophages and neutrophils upon *P. aeruginosa* infection, toxin-based (lipochlodronate, nitroreductase system) or genetic tools (morphants) are available to specifically deplete one or the other cell population. In the right panel, images show the efficiency of macrophage depletion by lipochlodronate (lipoCld) in *tg(mfap4:mCherry-F)* fish line (Moussouni et al., 2021). Survival curves of lipoCld-treated and non-treated embryos infected with PAO1 wild-type stain or PAO1 Δ*oprF* mutant are shown (Moussouni et al., 2021). Created with BioRender.com.

Microinjection in a closed compartment such as the hindbrain ventricle (HBV), otic vesicle, or tail muscle provides a local infection that facilitates live imaging. Notably, injection in HBV or muscle allowed to visualize in real time macrophages that migrate at the injection site to phagocytose bacteria (Moussouni et al., 2021) (**Figure 2**). Neutrophil recruitment has also been monitored upon HBV injection (McCarthy et al., 2017). Interestingly, lines of evidence of the development of *P*. *aeruginosa* microcolonies were reported upon HBV injection 24 hpi, which have been proposed as precursors of biofilm (Rocker et al., 2015). Accordingly, microcolonies were less frequent and of lower size and volume for a *psl* mutant, which is defective in the production of Psl exopolysaccharide and thus biofilm formation (Rocker et al., 2015). This study was, however, conducted on fixed and not live embryos. To further evaluate biofilm initiation *in vivo*, it would be of great interest to follow the formation of microcolonies in real time on live embryos over a period longer than 24 h.

The swim bladder shares similarities with the lung, with an air–epithelial interface that produces epithelial mucus, and localized microinjection in the swim bladder has been used to model mucosal mixed infection with *Candida albicans* and *P. aeruginosa* (Bergeron et al., 2017). Infection within the swim bladder is usually performed at 4 dpf, when the organ starts to inflate and is visible for microinjection.

### Bath Immersion

Microinjection of bacterial cells into zebrafish embryos requires specific expertise and is time consuming. Bath immersion, which is easier to handle, is a natural way of infection for aquatic bacterial pathogens (Rowe et al., 2014), but not for other pathogens. A lethal infection after bath immersion of 3 dpf healthy embryos with concentrated *P. aeruginosa* culture was reported in one report (Diaz-Pascual et al., 2017), but was not reproduced in other studies (Clatworthy et al., 2009; van Soest et al., 2011; Nogaret et al., 2021). Proteomic analysis indicated induction of hypoxia response, but no inflammatory response, in zebrafish embryos exposed to the immersion method, suggesting that healthy larvae suffered from a lack of oxygen when exposed to *P. aeruginosa* by static immersion (Diaz-Pascual et al., 2017). Moreover, the host response was also likely corresponding to a response against bacteria that are outside the fish or in contact with its skin (Diaz-Pascual et al., 2017).

An alternative infection route has been recently proposed with the bath immersion of tail-injured embryos (Poplimont et al., 2020; Nogaret et al., 2021). A rapid (within 24 h) bacterial dose-dependent mortality was observed when 2 dpf embryos were injured at the tail fin before static immersion with *P. aeruginosa* PAO1 strain. Moreover, a mutant strain known to be attenuated upon microinjection in the caudal vein is similarly attenuated upon bath infection of injured embryos (Nogaret et al., 2021), supporting the reliability of the bath model in assessing *P. aeruginosa* virulence. This infection mode, which could be considered as a model of wound infection, is of interest for drug screening (see below).

## Interplay of Pseudomonas aeruginosa With the Zebrafish Innate Immune System

Infection of zebrafish embryos by *P. aeruginosa* induces an acute infection and lethality of embryos, mostly within 24–30 hpi, suggesting that infection is cleared or controlled in surviving embryos. The embryo response to *P. aeruginosa* infection is strongly related to the initial concentration of infection bacteria. The zebrafish is relatively resistant to *Pseudomonas*, and injection of large inocula (above 1,000 bacteria per embryo) is required to induce host killing. Macrophages and neutrophils can rapidly phagocytose *P. aeruginosa*, suggesting that both phagocytic cell types play a role in protection against infection (Brannon et al., 2009; Clatworthy et al., 2009; Cafora et al., 2019). A *P. aeruginosa* clinical isolate from a CF patient microinjected into the duct of Cuvier was also predominantly found to be associated or engulfed by macrophages within 6 hpi (Kumar et al., 2018). **Figure 2** recapitulates some advantages of the zebrafish embryo model to address the role of phagocytic cells during *P. aeruginosa* infection.

Phagocytosis of *P. aeruginosa*, injected in the muscle or HBV, by recruited macrophages has been visualized in real time (Cafora et al., 2019; Moussouni et al., 2021) (**Figure 2**). Depletion of macrophages, through the use of *pu.1* morphants or lipochlodronate, increased the susceptibility of larvae to *P. aeruginosa* (Brannon et al., 2009; Belon et al., 2015; Moussouni et al., 2021) (**Figure 2**), supporting the role of macrophages in the clearance of *P. aeruginosa* during acute infection. Survival of infected embryos is reduced, in association with increased bacterial burden, in embryos defective for the mitochondrial superoxide dismutase 2 (*sod2* morphant), which contributes to generating oxidative stress in phagocytes (Peterman et al., 2015). In addition, the survival of infected embryos is largely reduced in the presence of bafilomycin, which inhibits host vacuolar ATPase, supporting the idea that acid stress within phagocytic cells is important for host defense (Moussouni et al., 2021).

The recruitment of neutrophils at the site of infection has also been observed (Diaz-Pascual et al., 2017; McCarthy et al., 2017). The effect of neutrophil depletion in the outcome of *P. aeruginosa* in zebrafish embryos has not been investigated, but defects in neutrophil migration greatly sensitize embryos to *P. aeruginosa* infections (Rosowski et al., 2016; Houseright et al., 2020). More precisely, embryos defective for Rac2 function, which have neutrophils that are unable to migrate, are highly susceptible to *P. aeruginosa* (Rosowski et al., 2016). Upon localized otic infection with *P. aeruginosa*, systemic activation and mobilization of neutrophils from hematopoietic tissues is also mediated by Cxcr2 signaling, a receptor enabling neutrophils to sense the IL8 chemokine (Deng et al., 2013). In addition, the increased susceptibility to *P. aeruginosa* infection observed in zebrafish deficient for *c3a.1*, which is homologous to the C3 component of human complement, is likely due to a neutrophil-intrinsic function of C3, possibly its ability to recruit neutrophils at the infection site (Houseright et al., 2020).

In parallel to the recruitment of phagocytic cells, *P. aeruginosa* triggers a potent proinflammatory response in zebrafish, with an increased expression of cytokines TNF-α and IL-β that was monitored by RT-qPCR (Clatworthy et al., 2009; Cafora et al., 2019). However, even though a correlation has been made between the intensity of the TNF-α response and the virulence of strains (Clatworthy et al., 2009), it is still unclear whether the mortality of embryos is due to a cytokine storm. An inflammatory response is also supported by global expression profile (see below).

Interestingly, the diguanylate cyclase SadC and the methyltransferase WarA, which interact with the LPS biosynthesis machinery of *P. aeruginosa* to modify the distribution of LPS O antigen, have been involved in neutrophil recruitment (McCarthy et al., 2017). *SadC* and *warA* mutants are slightly but significantly attenuated during zebrafish infection at early time post-infection (12 hpi), without being associated with a reduced bacterial load (McCarthy et al., 2017). During infection with *sadC* or *warA* mutant in HBV, more neutrophils were recruited to the site of infection, and a higher expression of the LPS-associated proinflammatory cytokine TNF-α was measured as compared to larvae infected with the wild-type strain, suggesting that SadC/WarA modifications of LPS mediate immune evasion *in vivo*.

Taken together, these studies show the strong contribution of zebrafish’s innate immune system in the outcome of *P. aeruginosa* infection and highlight the powerful tools available to decipher the interplay between pathogen and host components. These findings corroborate the role of innate immunity in humans with the increased sensibility of immunocompromised patients, such as neutropenic patients, to *P. aeruginosa* infection (Sadikot et al., 2005).

## *Pseudomonas aeruginosa* Factors Involved in Pathogenesis in Zebrafish

The zebrafish infection model has been used to assess the virulence of various *P. aeruginosa* mutant strains, thus allowing us to evaluate the role of different bacterial factors in this model. The mutants identified as attenuated in this model are summarized in **Table 1**. Of specific interest, several mutants are as virulent as a wild-type strain in macrophage-depleted embryos, indicating an interplay between these bacterial factors and phagocytes.

**Table 1:** *Pseudomonas aeruginosa* mutants attenuated in the zebrafish embryo infection model.

Gene name	Site of injection	References
*exsA* ^a^, *pscD* (T3SS)	CV, duct of Cuvier	Brannon et al. (2009); Clatworthy et al. (2009)
*oprF* ^a^	CV, HBV, immersion	Moussouni et al. (2021); Nogaret et al. (2021)
*mgtC* ^a^	CV	Belon et al. (2015)
*mvfR*, *lasR* (QS)	Duct of Cuvier	Clatworthy et al. (2009)
*phz1/2*	Duct of Cuvier	Chand et al. (2011)
*sarA*/*warA*	HBV	McCarthy et al. (2017)
*retS*, *gacS*, *phoR*, *copS*, *bqsS*, *kinB* (TCS)	Duct of Cuvier	Chand et al. (2011)
*PA2206* (LysR regulator)	CV	Reen et al. (2013)
*vreR*	CV	Llamas et al. (2009)

a Attenuation in a macrophage-dependent manner.

### T3SS, QS, and Other Classical Virulence Factors

A PA14 mutant lacking T3SS-structural protein PscD is attenuated upon microinjection into the yolk circulation valley at 2 dpf (Clatworthy et al., 2009). Accordingly, a PAK mutant strain lacking ExsA, which positively regulates T3SS, is also attenuated upon microinjection in the caudal vein of 2 dpf embryos (Brannon et al., 2009). The first effect of T3SS was reported between 4 and 8 hpi, when the PAK strain, but not an *exsA* mutant, began to proliferate despite an initial phase of bacterial clearance, probably by phagocytes (Brannon et al., 2009).

The virulence of *P. aeruginosa* strains lacking a functional T3SS can be restored upon phagocyte depletion, suggesting that T3SS influences virulence through its effects on phagocytes (Brannon et al., 2009). The dramatic dependence of *P. aeruginosa* on the T3SS to overcome normal phagocyte defenses in the absence of adaptive immunity suggests that the zebrafish may be a useful and relevant model to understand the details of T3SS–phagocyte interactions. The specific involvement of T3SS effectors during infection in the zebrafish model still remains to be investigated.

Two bacterial factors, the inner membrane protein MgtC and the outer membrane porin OprF, which are involved in the intramacrophage survival of *P. aeruginosa*, have been involved in the regulation of T3SS genes (Garai et al., 2019). Similar to T3SS mutants, *mgtC* and *oprF* mutants (in the PAO1 background) have been shown to be attenuated after microinjection in the caudal vein of 2 dpf embryos in a macrophage-dependent manner (Belon et al., 2015; Moussouni et al., 2021) (**Figure 2**). Attenuation of *oprF* mutant has also been reported upon infection by immersion of tail-injured embryos (Nogaret et al., 2021). Interestingly, the infection defect of *oprF* mutant in zebrafish embryo can be suppressed upon the addition of bafilomycin, an inhibitor of phagosomal acidification, which correlates with a preferential association of *oprF* mutant with acidified compartments in cultured macrophages (Moussouni et al., 2021).

LasR and MvfR are transcriptional regulators of QS that control many genes encoding virulence factors. *P. aeruginosa lasR* and *mvfR* mutants (PA14 background) are attenuated in zebrafish embryos infected at 2 dpf into the yolk circulation valley (Clatworthy et al., 2009).

Among other putative virulence genes tested, a *phz1/2* mutant defective in phenazine-1-carboxylic acid biosynthesis (whose main derivative is pyocyanin) was attenuated for virulence in the zebrafish embryo infection model, suggesting that pyocyanin is a critical virulence factor in this model (Chand et al., 2011), which may interfere with bacterial clearance by phagocytes. In contrast, mutants deficient for elastase production (*lasB*), flagellar motility (*flgK*), alginate (*algD*), or Pel exopolysaccharide (*pelA*) production were not attenuated during acute infection in zebrafish embryos (Chand et al., 2011).

### Two-Component Systems and Transcriptional Regulators

The role of 60 two-component sensors (PA14 genetic background) has been systematically tested with individual mutants in 50 hpf zebrafish embryos microinjected into the bloodstream through the yolk circulation valley. Six sensors that are required for *P. aeruginosa* virulence were identified (GasS, RetS, PhoR, CopS, BqsS, KinB) (Chand et al., 2011). A deeper analysis of *kinB* mutant showed that KinB is required for acute infection in zebrafish embryos and regulates a number of virulence-associated phenotypes, including QS, pyocyanin production, biofilm formation, and motility (Chand et al., 2011).

Extracytoplasmic function sigma factors are important signal-responsive regulatory proteins in *P. aeruginosa*, and a member of this family, σ^VreI^, promotes the transcription of secretion systems and secreted proteins. The VreR anti-sigma factor, involved in the regulation of σ^VreI^, is required for *P. aeruginosa* virulence in zebrafish embryos, possibly through the modulation of bacterial toxicity toward host cells (Llamas et al., 2009; Otero-Asman et al., 2020).

The non-classical LysR-type transcriptional regulator PA2206 is required for an effective oxidative stress response in *P. aeruginosa* (required for tolerance to H_2_O_2_ *in vitro*) and was found to be important for the lethality of zebrafish embryos injected at 26 hpf in the blood island (PAO1 derivative) (Reen et al., 2013). Whether the attenuated phenotype of PA2206 mutant in the zebrafish infection model is directly linked to its reduced tolerance to oxidative stress remains to be determined. In addition, the implication of phagocytic cells in the attenuated phenotype has not been investigated.

Cumulatively, the use of diverse mutated strains indicates that the relevance of *P. aeruginosa* factors, such as T3SS and QS, during pathogenesis in zebrafish embryo model is consistent with the results reported in acute models of mice infection (Rumbaugh et al., 1999; Vance et al., 2005; Kumar et al., 2009). In addition, virulence phenotypes in zebrafish corroborate clinical observations in humans, since T3SS has been shown to increase the infection risk and is associated with poor clinical outcomes (Hauser et al., 2002; Ledizet et al., 2012), similar to pyocyanin production upon bloodstream infections (Gupte et al., 2021). On the other hand, similar to what is observed in zebrafish, swimming motility and protease secretion have not been associated with increased pathogenicity and disease severity in humans (Ledizet et al., 2012; Gupte et al., 2021).

## Expression of Host and *Pseudomonas aeruginosa* Genes During Infection

The transparency of the zebrafish embryos can be used to monitor bacterial gene expression *in vivo* using strains with transcriptional fusions with fluorescent reporter genes. Expression *in vivo* of a *P. aeruginosa* gene regulated by the σ^VreI^ transcriptional factor was observed upon bacterial injection in HBV (Otero-Asman et al., 2020). How activation of σ^VreI^ in response to the host occurs remains to be investigated.

The use of a zebrafish embryo model also allows us to assess the global gene expression of both host and microbe in parallel. This provides a unique opportunity to investigate the molecular mechanisms underlying the interaction between the host’s innate immune system and the pathogen.

A global proteomic approach was used to track simultaneously *in vivo* the pathogen response and host immune response at 22 hpi using 3 dpf zebrafish larvae infected with *P. aeruginosa* PAO1 by immersion (without tail injury) or microinjection (Diaz-Pascual et al., 2017). Some zebrafish metabolic pathways, such as hypoxia response, as well as the integrin signaling pathway and angiogenesis, were exclusively enriched in the larvae exposed by static immersion. In contrast, inflammation mediated by chemokine and cytokine signaling pathways was exclusively enriched in the larvae exposed by injection. Important virulence factors from *P. aeruginosa*, involved in toxin production, T3SS, QS, and the production of extracellular polymeric substances, were enriched only after exposure by injection, which is consistent with the role of these factors during acute infection in this model (**Table 1**).

A dual host–pathogen transcriptomic analysis was also conducted at a later infection time (3 dpi) on embryos surviving infection using a CF clinical isolate (PASS1) microinjected into the duct of Cuvier at 2 dpf (Kumar et al., 2018). PASS1 displayed increased expression of an array of genes shown previously to be important in pathogenesis, including genes encoding pyoverdine biosynthesis, flagellin, non-hemolytic phospholipase C, proteases, superoxide dismutase, and fimbrial subunits. In addition, phosphate and iron acquisition genes are significantly upregulated in PASS1, suggesting that phosphate and iron are limiting nutrients within the zebrafish host. Regarding the host, proinflammatory genes as chemokine receptors, IL-1β, Toll-like receptors, and TNF receptor signaling family were activated. Transcriptional regulators of neutrophil and macrophage phagocytosis were also upregulated, highlighting phagocytosis as a key response mechanism to *P. aeruginosa* infection.

Real-time imaging using zebrafish transgenic lines carrying reporters of inflammatory genes and a cohort of clinical isolates could increase the relevance of these observations, notably by shedding light on the ability of strains from different origins to escape immune detection. In humans, host responses to chronic *P. aeruginosa* infections are indeed complex, ranging from vigorous inflammation ineffective at eradicating infecting bacteria, to relative host tolerance through a dampened activation of host immunity (Faure et al., 2018).

## Role of Host Factors During *Pseudomonas aeruginosa* Infection: The Case of CFTR

As indicated above, *P. aeruginosa* infections are a major cause of mortality and morbidity in patients with CF. The CFTR channel has a broad cellular distribution and CF affects multiple organs in humans including the lung, gastrointestinal tract, liver, male reproductive tract, and pancreas.

### CFTR in Zebrafish

The amino acid sequence of zebrafish CFTR (zCFTR) is 55% identical to human CFTR (hCFTR), and 42 out of 46 sites of mutations found in patients with CF are conserved in zCFTR. The structure of zCFTR was the first CFTR structure to be solved (Zhang and Chen, 2016; Zhang et al., 2017) and is essentially identical to the structure of hCFTR (Liu et al., 2017). Zebrafish *cftr* is expressed in the liver, kidney, spleen, and intestine, and *cftr* transcripts are detected in cells of the myeloid lineage that includes macrophages and neutrophils (Phennicie et al., 2010).

The role of CFTR in zebrafish was addressed by generating a fish line with frameshift mutation in the *cftr* gene (Navis et al., 2013; Liao et al., 2018). In zebrafish, left–right asymmetry requires cilia-driven fluid flow within the lumen of Kupffer’s vesicle (KV), and a reduced fluid secretion in *cftr* mutant impairs KV lumen expansion leading to defects in organ laterality (Navis et al., 2013). Due to male infertility (Liao et al., 2018), the lines have to be maintained at heterozygous stage, and homozygous embryos are screened based on the altered KV morphogenesis (Navis et al., 2013). Interestingly, deregulation of *cftr* function in zebrafish causes a phenotype that mirrors other defects present in the human disease such as severe pancreatic dysfunction (Navis and Bagnat, 2015) and hematopoietic defects (Sun et al., 2018), which may correlate with anemia presented by patients with CF.

Zebrafish embryos with *cftr*-loss-of-function (also called “CF embryos”) thus represent a promising model to study the implication of CFTR in innate immune response and mucin secretion. Another way to generate a CF zebrafish model, which has been used in the context of *P. aeruginosa* infection, is through the injection of morpholino that transiently knocks down *cftr* gene expression.

### *Pseudomonas aeruginosa* Infection in CF Zebrafish Embryos

CF zebrafish embryos (*cftr* morphants) have a 3.5-fold higher number of *P*. *aeruginosa* PA14 bacterial cells after 8 h of infection than control embryos (Phennicie et al., 2010). A similar pattern of differences in bacterial burden at early time post-infection was observed with a *P. aeruginosa* CF clinical isolate. However, no effect was reported at later times, and this difference early in the course of infection did not result in higher mortality in the *cftr* morphants compared with that of the control. On the other hand, in another study carried out with PAO1 strain microinjected in the duct of Cuvier at 48 hpf, the mortality of the CF embryos was slightly, but significantly, increased at 20 hpi comparative to control embryos (Cafora et al., 2019). Time laps analyses after HBV injection by confocal microscopy showed several microcolonies at 18 hpi (Rocker et al., 2015), and interestingly, the area of microcolonies formed in CF embryos was reported to be higher than in wild-type fish (Cafora et al., 2019). A deeper analysis of the formation of these microcolonies over a long period would be of interest to determine if they persist, increase in size and harbor biofilm characteristics, or are eliminated.

The production of reactive oxygen species is significantly dampened in *cftr* morphants compared with control embryos, and a reduction of neutrophil migration toward the injection site is observed in the case of local injection (HBV), supporting a link between CFTR and innate immune response (Phennicie et al., 2010). Moreover, CF embryos present a reduced proinflammatory immune response following bacterial infection in comparison with the wild-type, as shown by significantly reduced TNF-α and IL-1β responses (Phennicie et al., 2010; Cafora et al., 2019).

Taken together, these results indicate that zCFTR contributes, to a moderate extent, to the resistance against *P. aeruginosa* infection. This is likely linked to an alteration of the inflammatory response and could also be related to an alteration of the bactericidal action of innate immune cells (Bernut et al., 2019). Such findings corroborate a reduced clearance of *P. aeruginosa* in the CF mouse model and in patients with CF, which is in part linked to the altered ability of CF macrophages to properly control the inflammatory response and kill bacteria (Hartl et al., 2012). The results in zebrafish rely on a morphant-based CF model and it would be of interest to carry out *P. aeruginosa* infection with a mutated fish line, to strengthen the invalidation of the *cftr* gene.

## Validation of Anti-*Pseudomonas* Strategies in Zebrafish

Aside from being of interest as an infection model, the zebrafish embryo is also suitable for *in vivo* chemical screening (Zon and Peterson, 2005; Rennekamp and Peterson, 2015), with the advantage that permeability of the larvae allows the entry of small compounds added directly to the fish water. This model, which allows also to address drug toxicity (Eimon and Rubinstein, 2009), has been successfully used for drug testing in the context of infectious diseases (Bernut et al., 2014b). The mode of infection with bath immersion of cut-tailed embryos in 96-well plates is of particular interest for the screening of anti-infectious compounds.

### Test of Clinically Used Antibiotics

Treatment of infected embryos, microinjected at 50 hpf with PA14 in the circulation, with ciprofloxacin (50 µg/ml) or imipenem (50 µg/ml) added in the bath medium, could rescue embryos from lethality (Clatworthy et al., 2009). Protection with ciprofloxacin (50 µg/ml) was also observed with the infection mode of tail-injured embryos (Nogaret et al., 2021). In this case, an antibiotic was added in the bath 2 h after injury and bacterial immersion, at a time when the wound was closed. Interestingly, a significant protective effect, of lower amplitude, was also shown at a much lower dose of 1 µg/ml, supporting the pertinence of the model for drug testing.

### Validation of Novel Antibacterial Strategies

Phage therapy using a phage cocktail, found to be efficient to treat *P. aeruginosa* acute infections in mouse and *Galleria mellonella* larvae, was used in zebrafish embryos (Cafora et al., 2019; Cafora et al., 2020b). Phage therapy, done by injecting a phage cocktail in the yolk sac of PAO1-infected embryos, was shown to reduce lethality, bacterial burden, and the proinflammatory response caused by PAO1 infection at 20 hpi both in wild-type and CF zebrafish (Cafora et al., 2019). In addition, phages alone mitigate inflammation in wild-type and CF zebrafish by reducing the expression levels of proinflammatory cytokines and the neutrophilic recruitment to the infection site (Cafora et al., 2020a).

Anti-virulence strategies have emerged as attractive novel therapeutic approaches that would apply less selective pressure to develop resistance and better preserve microbiota than traditional antimicrobial therapy (Muhlen and Dersch, 2016). In this context, the zebrafish embryo is suitable for testing the efficacy of specific inhibitors of virulence factors that are important for *P. aeruginosa* infection in this model (**Table 1**). This is the case of QS, which has been proposed as an attractive target to fight *P. aeruginosa* infections using alternative therapies (Smith and Iglewski, 2003). The bath immersion model of injured embryos was used to show the anti-infective potential of a novel anti-QS compound, without exhibiting any toxicity (Nogaret et al., 2021). This compound is an antagonistic analog of C_4_-HSL (Furiga et al., 2016) that was not previously tested in an animal model. *In vitro*, C11 was shown to inhibit *P. aeruginosa* biofilm formation and reduce the expression of QS regulatory/regulated genes (Furiga et al., 2016). Further studies are required to determine how C11 addition *in vivo* reduces embryo mortality, which can be due to its ability to downregulate QS gene expression and impair virulence and/or to the fact that initiation of infection at the injured tail shares properties with the initiation of biofilm formation.

Taken together, these studies support the relevance of zebrafish as a first-intention vertebrate model to validate molecules that reduce *P. aeruginosa* pathogenicity and to perform drug screening. In addition, therapeutic molecules can also be tested in a CF context.

## Conclusion and Perspectives

**Figure 3:**
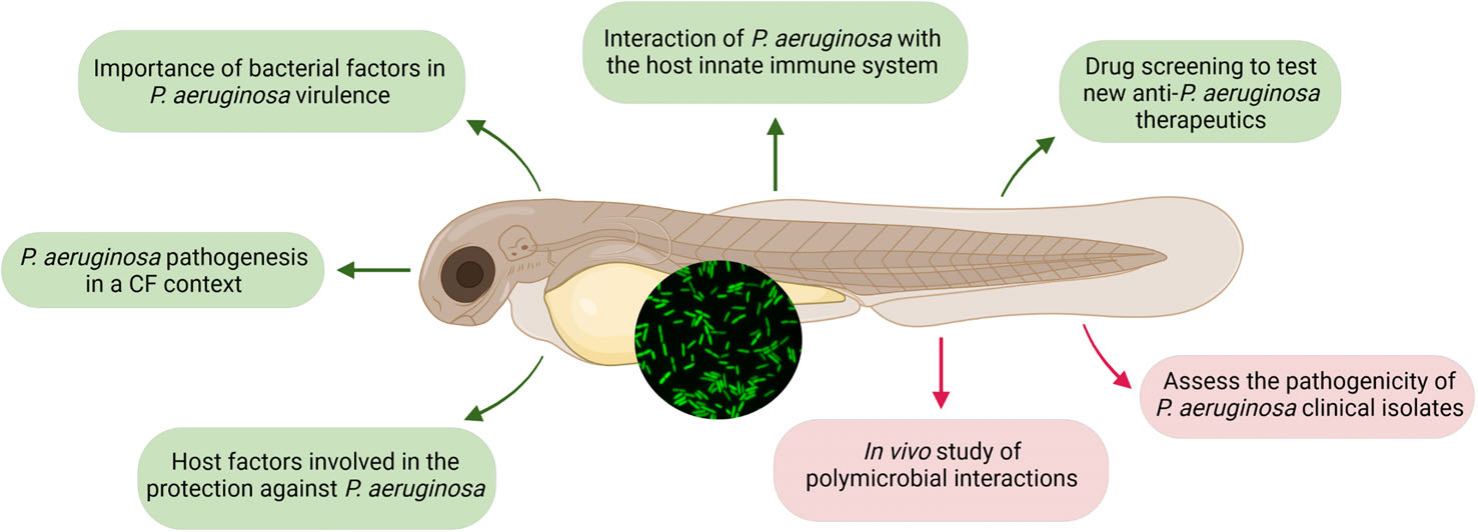
Insights from infecting zebrafish embryo with *Pseudomonas aeruginosa* (GFP-expressing bacteria are shown in the inserted picture). Insights presented in this review, using diverse infection routes, are represented in green, while new appealing perspectives that are clinically relevant are highlighted in red. Created with BioRender.com.

In the present review, we have recapitulated the multiple points of interest of using zebrafish embryos as an infection model for *P. aeruginosa* (**Figure 3**). This model appears as a potent first-intention vertebrate model to monitor *P. aeruginosa* pathogenesis and test novel therapeutic strategies. Importantly, this model offers unprecedented opportunities to investigate the role of phagocytic cells and inflammatory response during infection. Whereas the role of alveolar macrophages in internalization and early clearance of *P. aeruginosa* in mouse lung remains controversial (Kooguchi et al., 1998; Cheung et al., 2000), macrophages play a key role during *P. aeruginosa* infection in the zebrafish model, thus allowing to investigate how intraphagocytic stage contributes to dissemination, persistence, and susceptibility to drug treatment.

In addition to these multiple points of interest, zebrafish embryos also offer perspectives to study *P. aeruginosa* clinical isolates and polymicrobial infections (**Figure 3**). While reference laboratory strains causing acute infections (PAO1, PA14, and PAK) have been widely used in the zebrafish model to gain insights on the host–*P. aeruginosa* interaction *in vivo*, investigation of the pathogenesis of clinical isolates in this vertebrate model remains very scarce, with only two CF isolates used (Phennicie et al., 2010; Kumar et al., 2018). In the future, a broader and deeper analysis of the behavior of clinical acute or chronic strains would shed light on their pathogenesis and on the pertinence of the model to assess persistence strategies used by CF isolates, as well as investigate *in vivo* their ability to induce or reduce the production of proinflammatory cytokines.

Zebrafish embryo has been proposed as a novel vertebrate CF model, but the increased sensitivity of *cftr* morphants to *P. aeruginosa* infection remains moderate, suggesting that the model should be improved, possibly with the use of mutated fish lines. Importantly, zebrafish embryo has been reported as a suitable model for other opportunistic bacterial pathogens found in patients with CF, such as *Staphylococcus aureus*, *Burkholderia cenopacia*, and *Mycobacterium abscessus* (Prajsnar et al., 2008; Vergunst et al., 2010; Bernut et al., 2014a). The zebrafish embryo offers interesting opportunities for future studies of *P. aeruginosa* in the context of microbial communities, as well as in the immune response against polymicrobial infection (Bergeron et al., 2017). It is well known that multiple microbial species can interact together within a given microenvironment, particularly within mixed biofilms, and that it can contribute to increased disease severity during polymicrobial infections (Briaud et al., 2019). Co-infection of *C. albicans* and *P. aeruginosa* in zebrafish swim bladder, proposed as a suitable site to model mucosal lung, showed increased proliferation of both pathogens, as well as potentiated hyphal penetration through epithelial barriers and increased the host inflammatory response (Bergeron et al., 2017). This suggests *in vivo* cross talk that benefits both organisms to the detriment of the host and sheds light on the effect of cross-kingdom interactions on infection outcome. In addition, the use of gnotobiotic zebrafish colonized with *P. aeruginosa* also demonstrated that zebrafish provides opportunities to explore how habitat influences the establishment of microbiota, and how microbial dynamics *in vivo* affect host biology (Rawls et al., 2007).

## Author Contributions

SP and A-BB-P wrote the manuscript. SP drew the figures. All authors contributed to the article and approved the submitted version.

## Conflict of Interest

The authors declare that the research was conducted in the absence of any commercial or financial relationships that could be construed as a potential conflict of interest.

## Publisher’s Note

All claims expressed in this article are solely those of the authors and do not necessarily represent those of their affiliated organizations, or those of the publisher, the editors and the reviewers. Any product that may be evaluated in this article, or claim that may be made by its manufacturer, is not guaranteed or endorsed by the publisher.

## References

[B1] Arai H. (2011). Regulation and Function of Versatile Aerobic and Anaerobic Respiratory Metabolism in Pseudomonas Aeruginosa. Front. Microbiol. 2, 103. doi: 10.3389/fmicb.2011.00103 21833336 PMC3153056

[B2] Belon C. Soscia C. Bernut A. Laubier A. Bleves S. Blanc-Potard A. B. (2015). A Macrophage Subversion Factor Is Shared by Intracellular and Extracellular Pathogens. PloS Pathog. 11, e1004969. doi: 10.1371/journal.ppat.1004969 26080006 PMC4469704

[B3] Bergeron A. C. Seman B. G. Hammond J. H. Archambault L. S. Hogan D. A. Wheeler R. T. (2017). Candida Albicans and Pseudomonas Aeruginosa Interact To Enhance Virulence of Mucosal Infection in Transparent Zebrafish. Infect. Immun. 85, e00475. doi: 10.1128/IAI.00475-17 PMC5649025 28847848

[B4] Bernut A. Dupont C. Ogryzko N. V. Neyret A. Herrmann J. L. Floto R. A.. (2019). CFTR Protects Against Mycobacterium Abscessus Infection by Fine-Tuning Host Oxidative Defenses. Cell Rep. 26, 1828–182+. doi: 10.1016/j.celrep.2019.01.071 30759393 PMC7618368

[B5] Bernut A. Herrmann J. L. Kissa K. Dubremetz J. F. Gaillard J. L. Lutfalla G.. (2014a). Mycobacterium Abscessus Cording Prevents Phagocytosis and Promotes Abscess Formation. Proc. Natl. Acad. Sci. United States America. 111, E943–E952. doi: 10.1073/pnas.1321390111 PMC3956181 24567393

[B6] Bernut A. Le Moigne V. Lesne T. Lutfalla G. Herrmann J. L. Kremer L. (2014b). *In Vivo* Assessment of Drug Efficacy Against Mycobacterium Abscessus Using the Embryonic Zebrafish Test System. Antimicrob Agents Chemother. 58, 4054–4063. doi: 10.1128/AAC.00142-14 24798271 PMC4068527

[B7] Brannon M. K. Davis J. M. Mathias J. R. Hall C. J. Emerson J. C. Crosier P. S.. (2009). Pseudomonas Aeruginosa Type III Secretion System Interacts With Phagocytes to Modulate Systemic Infection of Zebrafish Embryos. Cell. Microbiol. 11, 755–768. doi: 10.1111/j.1462-5822.2009.01288.x 19207728 PMC2933946

[B8] Briaud P. Camus L. Bastien S. Doleans-Jordheim A. Vandenesch F. Moreau K. (2019). Coexistence With Pseudomonas Aeruginosa Alters Staphylococcus Aureus Transcriptome, Antibiotic Resistance and Internalization Into Epithelial Cells. Sci. Rep. 9, 16564. doi: 10.1038/s41598-019-52975-z 31719577 PMC6851120

[B9] Broncano-Lavado A. Santamaria-Corral G. Esteban J. Garcia-Quintanilla M. (2021). Advances in Bacteriophage Therapy Against Relevant MultiDrug-Resistant Pathogens. Antibiot (Basel). 10, 672. doi: 10.3390/antibiotics10060672 PMC8226639 34199889

[B10] Cafora M. Brix A. Forti F. Loberto N. Aureli M. Briani F. (2020a). Phages as Immunomodulators and Their Promising Use as Anti-Inflammatory Agents in a Cftr Loss-of-Function Zebrafish Model. J. Cystic Fibrosis. S1569–1993, 30927. doi: 10.1016/j.jcf.2020.11.017 33298374

[B11] Cafora M. Deflorian G. Forti F. Ferrari L. Binelli G. Briani F.. (2019). Phage Therapy Against Pseudomonas Aeruginosa Infections in a Cystic Fibrosis Zebrafish Model. Sci. Rep. 9, 1527. doi: 10.1038/s41598-018-37636-x 30728389 PMC6365511

[B12] Cafora M. Forti F. Briani F. Ghisotti D. Pistocchi A. (2020b). Phage Therapy Application to Counteract Pseudomonas Aeruginosa Infection in Cystic Fibrosis Zebrafish Embryos. J. Visualized Exp JoVE. 159. doi: 10.3791/61275 32478753

[B13] Chand N. S. Lee J. S. Clatworthy A. E. Golas A. J. Smith R. S. Hung D. T. (2011). The Sensor Kinase KinB Regulates Virulence in Acute Pseudomonas Aeruginosa Infection. J. Bacteriol. 193, 2989–2999. doi: 10.1128/JB.01546-10 21515773 PMC3133189

[B14] Cheung D. O. Halsey K. Speert D. P. (2000). Role of Pulmonary Alveolar Macrophages in Defense of the Lung Against Pseudomonas Aeruginosa. Infect. Immun. 68, 4585–4592. doi: 10.1128/IAI.68.8.4585-4592.2000 10899859 PMC98382

[B15] Clatworthy A. E. Lee J. S. Leibman M. Kostun Z. Davidson A. J. Hung D. T. (2009). Pseudomonas Aeruginosa Infection of Zebrafish Involves Both Host and Pathogen Determinants. Infect. Immun. 77, 1293–1303. doi: 10.1128/IAI.01181-08 19168742 PMC2663173

[B16] de Bentzmann S. Plesiat P. (2011). The Pseudomonas Aeruginosa Opportunistic Pathogen and Human Infections. Environ. Microbiol. 13, 1655–1665. doi: 10.1111/j.1462-2920.2011.02469.x 21450006

[B17] Del Porto P. Cifani N. Guarnieri S. Di Domenico E. G. Mariggio M. A. Spadaro F.. (2011). Dysfunctional CFTR Alters the Bactericidal Activity of Human Macrophages Against Pseudomonas Aeruginosa. PloS One 6, e19970. doi: 10.1371/journal.pone.0019970 21625641 PMC3097223

[B18] Deng Q. Sarris M. Bennin D. A. Green J. M. Herbomel P. Huttenlocher A. (2013). Localized Bacterial Infection Induces Systemic Activation of Neutrophils Through Cxcr2 Signaling in Zebrafish. J. Leukocyte Biol. 93, 761–769. doi: 10.1189/jlb.1012534 23475575 PMC4050646

[B19] Diaz-Pascual F. Ortiz-Severin J. Varas M. A. Allende M. L. Chavez F. P. (2017). *In Vivo* Host-Pathogen Interaction as Revealed by Global Proteomic Profiling of Zebrafish Larvae. Front. Cell. Infect. Microbiol. 7. doi: 10.3389/fcimb.2017.00334 PMC5524664 28791256

[B20] Di A. Brown M. E. Deriy L. V. Li C. Szeto F. L. Chen Y.. (2006). CFTR Regulates Phagosome Acidification in Macrophages and Alters Bactericidal Activity. Nat. Cell Biol. 8, 933–944. doi: 10.1038/ncb1456 16921366

[B21] Eimon P. M. Rubinstein A. L. (2009). The Use of *In Vivo* Zebrafish Assays in Drug Toxicity Screening. Expert Opin. Drug Met. 5, 393–401. doi: 10.1517/17425250902882128 19368493

[B22] Faure E. Kwong K. Nguyen D. (2018). Pseudomonas Aeruginosa in Chronic Lung Infections: How to Adapt Within the Host? Front. Immunol. 9. doi: 10.3389/fimmu.2018.02416 PMC6204374 30405616

[B23] Furiga A. Lajoie B. El Hage S. Baziard G. Roques C. (2016). Impairment of Pseudomonas Aeruginosa Biofilm Resistance to Antibiotics by Combining the Drugs With a New Quorum-Sensing Inhibitor. Antimicrob Agents Chemother. 60, 1676–1686. doi: 10.1128/AAC.02533-15 PMC4775964 26711774

[B24] Garai P. Berry L. Moussouni M. Bleves S. Blanc-Potard A. B. (2019). Killing From the Inside: Intracellular Role of T3SS in the Fate of Pseudomonas Aeruginosa Within Macrophages Revealed by mgtC and oprF Mutants. PloS Pathog. 15, e1007812. doi: 10.1371/journal.ppat.1007812 31220187 PMC6586356

[B25] Gellatly S. L. Hancock R. E. W. (2013). Pseudomonas Aeruginosa: New Insights Into Pathogenesis and Host Defenses. Pathog. Dis. 67, 159–173. doi: 10.1111/2049-632X.12033 23620179

[B26] Gomes M. C. Mostowy S. (2020). The Case for Modeling Human Infection in Zebrafish. Trends Microbiol. 28, 10–18. doi: 10.1016/j.tim.2019.08.005 31604611

[B27] Gupte A. Jyot J. Ravi M. Ramphal R. (2021). High Pyocyanin Production and non-Motility of Pseudomonas Aeruginosa Isolates are Correlated With Septic Shock or Death in Bacteremic Patients. PloS One 16, e0253259. doi: 10.1371/journal.pone.0253259 34115807 PMC8195364

[B28] Hartl D. Gaggar A. Bruscia E. Hector A. Marcos V. Jung A.. (2012). Innate Immunity in Cystic Fibrosis Lung Disease. J. Cystic Fibrosis Off. J. Eur. Cystic Fibrosis Soc. 11, 363–382. doi: 10.1016/j.jcf.2012.07.003 22917571

[B29] Hauser A. R. (2009). The Type III Secretion System of Pseudomonas Aeruginosa: Infection by Injection. Nat. Rev. Microbiol. 7, 654–665. doi: 10.1038/nrmicro2199 19680249 PMC2766515

[B30] Hauser A. R. Cobb E. Bodi M. Mariscal D. Valles J. Engel J. N.. (2002). Type III Protein Secretion is Associated With Poor Clinical Outcomes in Patients With Ventilator-Associated Pneumonia Caused by Pseudomonas Aeruginosa. Crit. Care Med. 30, 521–528. doi: 10.1097/00003246-200203000-00005 11990909

[B31] Hernandez Y. L. Yero D. Pinos-Rodriguez J. M. Gibert I. (2015). Animals Devoid of Pulmonary System as Infection Models in the Study of Lung Bacterial Pathogens. Front. Microbiol. 6. doi: 10.3389/fmicb.2015.00038 PMC4316775 25699030

[B32] Houseright R. A. Rosowski E. E. Lam P. Y. Tauzin S. J. M. Mulvaney O. Dewey C. N.. (2020). Cell Type Specific Gene Expression Profiling Reveals a Role for Complement Component C3 in Neutrophil Responses to Tissue Damage. Sci. Rep. 10, 15716. doi: 10.1038/s41598-020-72750-9 32973200 PMC7518243

[B33] Kaito C. Murakami K. Imai L. Furuta K. (2020). Animal Infection Models Using non-Mammals. Microbiol. Immunol. 64, 585–592. doi: 10.1111/1348-0421.12834 32757288 PMC7590188

[B34] Klockgether J. Tummler B. (2017). Recent Advances in Understanding Pseudomonas Aeruginosa as a Pathogen. F1000Res. 6, 1261. doi: 10.12688/f1000research.10506.1 28794863 PMC5538032

[B35] Kooguchi K. Hashimoto S. Kobayashi A. Kitamura Y. Kudoh I. Wiener-Kronish J.. (1998). Role of Alveolar Macrophages in Initiation and Regulation of Inflammation in Pseudomonas Aeruginosa Pneumonia. Infect. Immun. 66, 3164–3169. doi: 10.1128/IAI.66.7.3164-3169.1998 9632581 PMC108328

[B36] Kroken A. R. Chen C. K. Evans D. J. Yahr T. L. Fleiszig S. M. J. (2018). The Impact of ExoS on Pseudomonas Aeruginosa Internalization by Epithelial Cells Is Independent of fleQ and Correlates With Bistability of Type Three Secretion System Gene Expression. mBio. 9, 00668. doi: 10.1128/mBio.00668-18 PMC5930308 29717012

[B37] Kumar R. Chhibber S. Harjai K. (2009). Quorum Sensing is Necessary for the Virulence of Pseudomonas Aeruginosa During Urinary Tract Infection. Kidney Int. 76, 286–292. doi: 10.1038/ki.2009.183 19494801

[B38] Kumar S. S. Tandberg J. I. Penesyan A. Elbourne L. D. H. Suarez-Bosche N. Don E.. (2018). Dual Transcriptomics of Host-Pathogen Interaction of Cystic Fibrosis Isolate Pseudomonas Aeruginosa PASS1 With Zebrafish. Front. Cell. Infect. Microbiol. 8, 406. doi: 10.3389/fcimb.2018.00406 30524971 PMC6262203

[B39] Lamberti Y. Surmann K. (2021). The Intracellular Phase of Extracellular Respiratory Tract Bacterial Pathogens and its Role on Pathogen-Host Interactions During Infection. Curr. Opin. Infect. Dis. 34, 197–205. doi: 10.1097/QCO.0000000000000727 33899754

[B40] Ledizet M. Murray T. S. Puttagunta S. Slade M. D. Quagliarello V. J. Kazmierczak B. I. (2012). The Ability of Virulence Factor Expression by Pseudomonas Aeruginosa to Predict Clinical Disease in Hospitalized Patients. PloS One 7, e49578. doi: 10.1371/journal.pone.0049578 23152923 PMC3495863

[B41] Liao H. Chen Y. Li Y. Xue S. Liu M. Lin Z.. (2018). CFTR is Required for the Migration of Primordial Germ Cells During Zebrafish Early Embryogenesis. Reprod. 156, 261–268. doi: 10.1530/REP-17-0681 PMC6106808 29930176

[B42] Linnerz T. Hall C. J. (2020). The Diverse Roles of Phagocytes During Bacterial and Fungal Infections and Sterile Inflammation: Lessons From Zebrafish. Front. Immunol. 11, 1094. doi: 10.3389/fimmu.2020.01094 32582182 PMC7289964

[B43] Liu F. Zhang Z. Csanady L. Gadsby D. C. Chen J. (2017). Molecular Structure of the Human CFTR Ion Channel. Cell. 169, 85–95 e88. doi: 10.1016/j.cell.2017.02.024 28340353

[B44] Llamas M. A. van der Sar A. M. (2014). Assessing Pseudomonas Virulence With Nonmammalian Host: Zebrafish. Methods Mol. Biol. 1149, 709–721. doi: 10.1007/978-1-4939-0473-0_55 24818945

[B45] Llamas M. A. van der Sar A. Chu B. C. Sparrius M. Vogel H. J. Bitter W. (2009). A Novel Extracytoplasmic Function (ECF) Sigma Factor Regulates Virulence in Pseudomonas Aeruginosa. PloS Pathog. 5, e1000572. doi: 10.1371/journal.ppat.1000572 19730690 PMC2729926

[B46] Lorenz A. Pawar V. Haussler S. Weiss S. (2016). Insights Into Host-Pathogen Interactions From State-of-the-Art Animal Models of Respiratory Pseudomonas Aeruginosa Infections. FEBS Lett. 590, 3941–3959. doi: 10.1002/1873-3468.12454 27730639

[B47] Masud S. Torraca V. Meijer A. H. (2017). Modeling Infectious Diseases in the Context of a Developing Immune System. Curr. Topics Dev. Biol. 124, 277–329. doi: 10.1016/bs.ctdb.2016.10.006 28335862

[B48] McCarthy R. R. Mazon-Moya M. J. Moscoso J. A. Hao Y. Lam J. S. Bordi C.. (2017). Cyclic-Di-GMP Regulates Lipopolysaccharide Modification and Contributes to Pseudomonas Aeruginosa Immune Evasion. Nat. Microbiol. 2, 17027. doi: 10.1038/nmicrobiol.2017.27 28263305 PMC5341770

[B49] Mittal R. Lisi C. V. Kumari H. Grati M. Blackwelder P. Yan D.. (2016). Otopathogenic Pseudomonas Aeruginosa Enters and Survives Inside Macrophages. Front. Microbiol. 7, 1828. doi: 10.3389/fmicb.2016.01828 27917157 PMC5114284

[B50] Moussouni M. Berry L. Sipka T. Nguyen-Chi M. Blanc-Potard A. B. (2021). Pseudomonas Aeruginosa OprF Plays a Role in Resistance to Macrophage Clearance During Acute Infection. Sci. Rep. 11, 359. doi: 10.1038/s41598-020-79678-0 33432030 PMC7801371

[B51] Muhlen S. Dersch P. (2016). Anti-Virulence Strategies to Target Bacterial Infections. Curr. Top. Microbiol. Immunol. 398, 147–183. doi: 10.1007/82_2015_490 26942418

[B52] Navis A. Bagnat M. (2015). Loss of Cftr Function Leads to Pancreatic Destruction in Larval Zebrafish. Dev. Biol. 399, 237–248. doi: 10.1016/j.ydbio.2014.12.034 25592226 PMC4765326

[B53] Navis A. Marjoram L. Bagnat M. (2013). Cftr Controls Lumen Expansion and Function of Kupffer's Vesicle in Zebrafish. Dev. 140, 1703–1712. doi: 10.1242/dev.091819 PMC3621488 23487313

[B54] Nogaret P. El Garah F. Blanc-Potard A. B. (2021). A Novel Infection Protocol in Zebrafish Embryo to Assess Pseudomonas Aeruginosa Virulence and Validate Efficacy of a Quorum Sensing Inhibitor *In Vivo* . Pathog. 10, 401. doi: 10.3390/pathogens10040401 PMC8065929 33805384

[B55] Otero-Asman J. R. Quesada J. M. Jim K. K. Ocampo-Sosa A. Civantos C. Bitter W.. (2020). The Extracytoplasmic Function Sigma Factor Sigma(VreI) is Active During Infection and Contributes to Phosphate Starvation-Induced Virulence of Pseudomonas Aeruginosa. Sci. Rep. 10, 3139. doi: 10.1038/s41598-020-60197-x 32081993 PMC7035377

[B56] Pang Z. Raudonis R. Glick B. R. Lin T. J. Cheng Z. Y. (2019). Antibiotic Resistance in Pseudomonas Aeruginosa: Mechanisms and Alternative Therapeutic Strategies. Biotechnol. Adv. 37, 177–192. doi: 10.1016/j.biotechadv.2018.11.013 30500353

[B57] Peterman E. M. Sullivan C. Goody M. F. Rodriguez-Nunez I. Yoder J. A. Kim C. H. (2015). Neutralization of Mitochondrial Superoxide by Superoxide Dismutase 2 Promotes Bacterial Clearance and Regulates Phagocyte Numbers in Zebrafish. Infect. Immun. 83, 430–440. doi: 10.1128/IAI.02245-14 25385799 PMC4288898

[B58] Phennicie R. T. Sullivan M. J. Singer J. T. Yoder J. A. Kim C. H. (2010). Specific Resistance to Pseudomonas Aeruginosa Infection in Zebrafish is Mediated by the Cystic Fibrosis Transmembrane Conductance Regulator. Infect. Immun. 78, 4542–4550. doi: 10.1128/IAI.00302-10 20732993 PMC2976322

[B59] Poplimont H. Georgantzoglou A. Boulch M. Walker H. A. Coombs C. Papaleonidopoulou F.. (2020). Neutrophil Swarming in Damaged Tissue Is Orchestrated by Connexins and Cooperative Calcium Alarm Signals. Curr. Biol. Cb. 30, 2761–2776 e2767. doi: 10.1016/j.cub.2020.05.030 32502410 PMC7372224

[B60] Prajsnar T. K. Cunliffe V. T. Foster S. J. Renshaw S. A. (2008). A Novel Vertebrate Model of Staphylococcus Aureus Infection Reveals Phagocyte-Dependent Resistance of Zebrafish to non-Host Specialized Pathogens. Cell. Microbiol. 10, 2312–2325. doi: 10.1111/j.1462-5822.2008.01213.x 18715285

[B61] Rahme L. G. Tan M. W. Le L. Wong S. M. Tompkins R. G. Calderwood S. B.. (1997). Use of Model Plant Hosts to Identify Pseudomonas Aeruginosa Virulence Factors. Proc. Natl. Acad. Sci. United States America. 94, 13245–13250. doi: 10.1073/pnas.94.24.13245 PMC24294 9371831

[B62] Rawls J. F. Mahowald M. A. Goodman A. L. Trent C. M. Gordon J. I. (2007). *In Vivo* Imaging and Genetic Analysis Link Bacterial Motility and Symbiosis in the Zebrafish Gut. Proc. Natl. Acad. Sci. United States America. 104, 7622–7627. doi: 10.1073/pnas.0702386104 PMC1855277 17456593

[B63] Reen F. J. Haynes J. M. Mooij M. J. O'Gara F. (2013). A non-Classical LysR-Type Transcriptional Regulator PA2206 is Required for an Effective Oxidative Stress Response in Pseudomonas Aeruginosa. PloS One 8, e54479. doi: 10.1371/journal.pone.0054479 23382903 PMC3557286

[B64] Rennekamp A. J. Peterson R. T. (2015). 15 Years of Zebrafish Chemical Screening. Curr. Opin. In Chem. Biol. 24, 58–70. doi: 10.1016/j.cbpa.2014.10.025 25461724 PMC4339096

[B65] Rocker A. J. Weiss A. R. Lam J. S. Van Raay T. J. Khursigara C. M. (2015). Visualizing and Quantifying Pseudomonas Aeruginosa Infection in the Hindbrain Ventricle of Zebrafish Using Confocal Laser Scanning Microscopy. J. Microbiol. Methods 117, 85–94. doi: 10.1016/j.mimet.2015.07.013 26188283

[B66] Rosowski E. E. (2020). Illuminating Macrophage Contributions to Host-Pathogen Interactions *In Vivo*: The Power of Zebrafish. Infect. And Immun. 88, e00906. doi: 10.1128/IAI.00906-19 32179583 PMC7309627

[B67] Rosowski E. E. Deng Q. Keller N. P. Huttenlocher A. (2016). Rac2 Functions in Both Neutrophils and Macrophages To Mediate Motility and Host Defense in Larval Zebrafish. J. Immunol. 197, 4780–4790. doi: 10.4049/jimmunol.1600928 27837107 PMC5367389

[B68] Rowe H. M. Withey J. H. Neely M. N. (2014). Zebrafish as a Model for Zoonotic Aquatic Pathogens. Dev. And Comp. Immunol. 46, 96–107. doi: 10.1016/j.dci.2014.02.014 24607289 PMC4096445

[B69] Rumbaugh K. P. Griswold J. A. Hamood A. N. (1999). Contribution of the Regulatory Gene lasR to the Pathogenesis of Pseudomonas Aeruginosa Infection of Burned Mice. J. Of Burn Care Rehabil. 20, 42–49. doi: 10.1097/00004630-199901001-00008 9934636

[B70] Sadikot R. T. Blackwell T. S. Christman J. W. Prince A. S. (2005). Pathogen-Host Interactions in Pseudomonas Aeruginosa Pneumonia. Am. J. Of Respir. And Crit. Care Med. 171, 1209–1223. doi: 10.1164/rccm.200408-1044SO 15695491 PMC2718459

[B71] Smith R. S. Iglewski B. H. (2003). Pseudomonas Aeruginosa Quorum Sensing as a Potential Antimicrobial Target. J. Clin. Invest. 112, 1460–1465. doi: 10.1172/JCI200320364 14617745 PMC259138

[B72] Sun H. Wang Y. Zhang J. Chen Y. Liu Y. Lin Z.. (2018). CFTR Mutation Enhances Dishevelled Degradation and Results in Impairment of Wnt-Dependent Hematopoiesis. Cell Death Dis. 9, 275. doi: 10.1038/s41419-018-0311-9 29449653 PMC5833403

[B73] Tacconelli E. Carrara E. Savoldi A. Harbarth S. Mendelson M. Monnet D. L.. (2018). Discovery, Research, and Development of New Antibiotics: The WHO Priority List of Antibiotic-Resistant Bacteria and Tuberculosis. Lancet Infect. Dis. 18, 318–327. doi: 10.1016/S1473-3099(17)30753-3 29276051

[B74] Torraca V. Masud S. Spaink H. P. Meijer A. H. (2014). Macrophage-Pathogen Interactions in Infectious Diseases: New Therapeutic Insights From the Zebrafish Host Model. Dis. Models Mech. 7, 785–797. doi: 10.1242/dmm.015594 PMC4073269 24973749

[B75] Torraca V. Mostowy S. (2018). Zebrafish Infection: From Pathogenesis to Cell Biology. Trends Cell Biol. 28, 143–156. doi: 10.1016/j.tcb.2017.10.002 29173800 PMC5777827

[B76] Vance R. E. Rietsch A. Mekalanos J. J. (2005). Role of the Type III Secreted Exoenzymes S, T, and Y in Systemic Spread of Pseudomonas Aeruginosa PAO1 *In Vivo* . Infect. Immun. 73, 1706–1713. doi: 10.1128/IAI.73.3.1706-1713.2005 15731071 PMC1064930

[B77] van Soest J. J. Stockhammer O. W. Ordas A. Bloemberg G. V. Spaink H. P. Meijer A. H. (2011). Comparison of Static Immersion and Intravenous Injection Systems for Exposure of Zebrafish Embryos to the Natural Pathogen Edwardsiella Tarda. BMC Immunol. 12, 58. doi: 10.1186/1471-2172-12-58 22003892 PMC3206475

[B78] Vergunst A. C. Meijer A. H. Renshaw S. A. O'Callaghan D. (2010). Burkholderia Cenocepacia Creates an Intramacrophage Replication Niche in Zebrafish Embryos, Followed by Bacterial Dissemination and Establishment of Systemic Infection. Infect. Immun. 78, 1495–1508. doi: 10.1128/IAI.00743-09 20086083 PMC2849400

[B79] Zhang Z. Chen J. (2016). Atomic Structure of the Cystic Fibrosis Transmembrane Conductance Regulator. Cell. 167, 1586–1597 e1589. doi: 10.1016/j.cell.2016.11.014 27912062

[B80] Zhang Y. Li X. Grassme H. Doring G. Gulbins E. (2010). Alterations in Ceramide Concentration and pH Determine the Release of Reactive Oxygen Species by Cftr-Deficient Macrophages on Infection. J. Immunol. 184, 5104–5111. doi: 10.4049/jimmunol.0902851 20351190

[B81] Zhang Z. Liu F. Chen J. (2017). Conformational Changes of CFTR Upon Phosphorylation and ATP Binding. Cell. 170, 483–491 e488. doi: 10.1016/j.cell.2017.06.041 28735752

[B82] Zon L. I. Peterson R. T. (2005). *In Vivo* Drug Discovery in the Zebrafish. Nat. Rev. Drug discovery. 4, 35–44. doi: 10.1038/nrd1606 15688071

